# Early-life reproductive success predicts lifespan: intrinsic quality, not reproductive trade-offs, explains heterogeneity in a short-lived vertebrate

**DOI:** 10.1098/rstb.2025.0324

**Published:** 2026-11-12

**Authors:** Silvia Cattelan, Dario R. Valenzano

**Affiliations:** ^1^ Leibniz Institute on Aging, Fritz-Lipmann Institute (FLI), Jena, Germany; ^2^ Friedrich Schiller University, Jena, Germany

**Keywords:** ageing, lifespan, causal inference, killifish, evolution, prediction, reproductive biology

## Abstract

Individuals of the same species often display extensive lifespan heterogeneity. However, the factors underlying such heterogeneity remain largely elusive. Here, we examined whether lifespan variation in the naturally short-lived turquoise killifish (*Nothobranchius furzeri*), an annual teleost with a limited reproductive window, is caused by reproductive trade-offs. To examine the individual covariation in reproduction and lifespan, we focused on males and performed *in vitro* fertilization using ejaculates collected longitudinally throughout life. We found that lifespan and reproduction do not trade off: early-life reproductive success positively predicts male lifespan and reflects intrinsic quality (IQ). A causal model, where IQ affects both survival and reproduction, recapitulates the empirical patterns. Together, our results show inter-individual variation in IQ explains lifespan heterogeneity in a short-lived vertebrate, challenging the view that reproductive costs drive inter-individual lifespan variation.

This article is part of the theme issue ‘Developmental circuits in ageing’.

## 1. Introduction

Heterogeneity in lifespan among individuals of the same species has been attributed to several causes, including survival–reproduction trade-offs, stochastic variation in early-life exposures, biotic and abiotic conditions and inter-individual differences in intrinsic quality (IQ; reviewed in [1]). Classical life-history theory, grounded in the assumption of limited resources, posits that organisms strategically allocate resources between reproduction and somatic maintenance [2], thereby generating much of the observed variation in lifespan both within and between species [3–5]. Under this framework, greater investment in rapid growth and early-life reproduction is expected to come at the cost of somatic maintenance, leading to accelerated senescence and reduced longevity [3,4]. Although stochastic processes undoubtedly contribute to individual life histories [6], work in genetically identical systems indicates that early-life variability is not expressed as irreducible noise but can instead be amplified and stabilized into persistent physiological states that are predictive of lifespan. In isogenic *Caenorhabditis elegans* populations, stochastic differences in the early expression of stress- and ageing-associated genes predict remaining lifespan, revealing stable inter-individual differences that emerge despite uniform genetic and environmental conditions [7]. Similarly, recent single-organism transcriptomic analyses demonstrate that early transcriptional heterogeneity can become canalized into coherent gene-expression programmes that persist across the lifespan and strongly predict survival outcomes [8]. Together, these findings suggest that stochasticity primarily acts by shaping durable differences in intrinsic individual state, rather than constituting an alternative, age-dependent explanation for lifespan variation. Inter-individual variation in IQ, here defined as stable, individual-specific differences that affect both reproductive and somatic performance, potentially owing to genetic, developmental or physiological factors [9,10], can generate positive correlations between reproduction and survival [11–14]. Within this framework, a central and still unresolved question is to what extent does heterogeneity in lifespan reflect differences in resource allocation strategies versus persistent differences in intrinsic individual quality.

Sex-specific life-history strategies provide a powerful context in which to address this question. Under classical life-history models, elevated male investment in early-life reproduction, in terms of conspicuous coloration, courtship behaviour, male–male competition and even sperm competition, is expected to incur costs to somatic maintenance, generating a negative association between reproduction and survival and favouring a ‘live fast–die young’ strategy [15,16]. However, another line of theoretical and experimental work suggests that sexual selection on males may instead favour traits that enhance both reproductive success and survival [17], potentially selecting for overall individual quality [18]. Under this framework, high-quality males are expected to both reproduce more and live longer, generating positive covariation between reproductive output and lifespan [19]. Importantly, such positive associations do not necessarily imply a strictly genetic basis, but may also arise from condition-dependent effects, environmental variation or their interaction with genetic factors, particularly under favourable environmental conditions [20]. Despite its central importance for understanding the origins of lifespan heterogeneity, empirical evidence addressing these alternative predictions in males remains scarce (reviewed in [21]).

The turquoise killifish (*Nothobranchius furzeri*) has evolved in ephemeral habitats where individuals can mate within a narrow time window, defined by seasonal water availability. Males show a strong and rapid decline in reproductive functions [22,23] despite their extremely short lifespan of 4–8 months [24]. Alternative life-history strategies among males may explain both the considerable variation in lifespan among individuals [24–26] and reproductive ageing [22,23], through the selective disappearance of ‘live fast−die young’ individuals from the population.

To investigate whether the variation in turquoise killifish lifespan reflects differential investment in reproduction among males, we used a longitudinal experimental design in which we measured male reproductive success after controlled *in vitro* fertilizations (IVF) at three different time points during lifespan and recorded the age of death. Under the trade-off model, we expect to find a negative association between survival and reproductive success, especially in early life. Alternatively, in the absence of obvious trade-offs, individual quality may affect both individual lifespan and reproduction, resulting in positive covariation between reproductive success and lifespan.

## 2. Results

### (a) Male reproductive success decreases with age and does not trade-off with lifespan

To investigate whether the variation in lifespan among male turquoise killifish (figure 1a) reflects differential investment in reproduction, we first performed IVFs at three different ages during male lifespan: at young (9 weeks post-hatching (wph), 100% population alive), middle (15 wph, 85% population alive) and old (20 wph, 60% population alive) age (figure 1b), by keeping female age constant throughout the experiment. As a measure of reproductive success, we counted the number of fertilized eggs (i.e. fertilization rate) and the number of embryos that survived after 72 hours after fertilization (i.e. embryo survival). Here, we present the results for embryo survival since fertilization rate largely mirrors the patterns observed in embryo survival (see the supplementary material, figure S1). First, confirming previous work [22], we found that male age significantly affected embryo survival (***χ***^2^ = 26.805, *p* = 1.512e–06). Old males produced embryos with significantly lower survival compared with young males (*z* = 4.572, *p* < 0.0001; figure 1c). Moreover, we found that embryo survival significantly declines already at mid-age (*z* = 3.764, *p* = 0.0005) but did not decrease further at old age (*z* = 1.986, *p* = 0.1155; figure 1c). Second, to examine potential reproduction-survival trade-offs, we estimated posterior predictive distributions of the relationship between lifespan and embryo survival at the three different ages. We fitted Bayesian binomial models [27,28] with a logit link function modelling the log-odds of embryo survival (i.e. the logit of the embryo survival probability) as a linear function of log-transformed lifespan (see equation (4.1)). Contrary to the trade-off expectation, we found no negative correlation between individual reproductive success and lifespan (figure 1c). Moreover, posterior estimates indicate that individuals with higher reproductive success at young age live longer, that is early-life reproductive success positively covaries with individual age at death (*β*_mean_ = 1.07; 0.51–1.67, 95% credible intervals), whereas there is no correlation between individual survival and reproductive outcome when sampling fathers at mid (*β*_mean_ = −0.15; −0.95 to −0.65, 95% credible intervals) and old age (*β*_mean_ = −0.14; −2.98 to −2.33, 95% credible intervals; figure 1c). These results indicate a positive association between embryo survival and lifespan at young age but not later in life.

**Figure 1.**
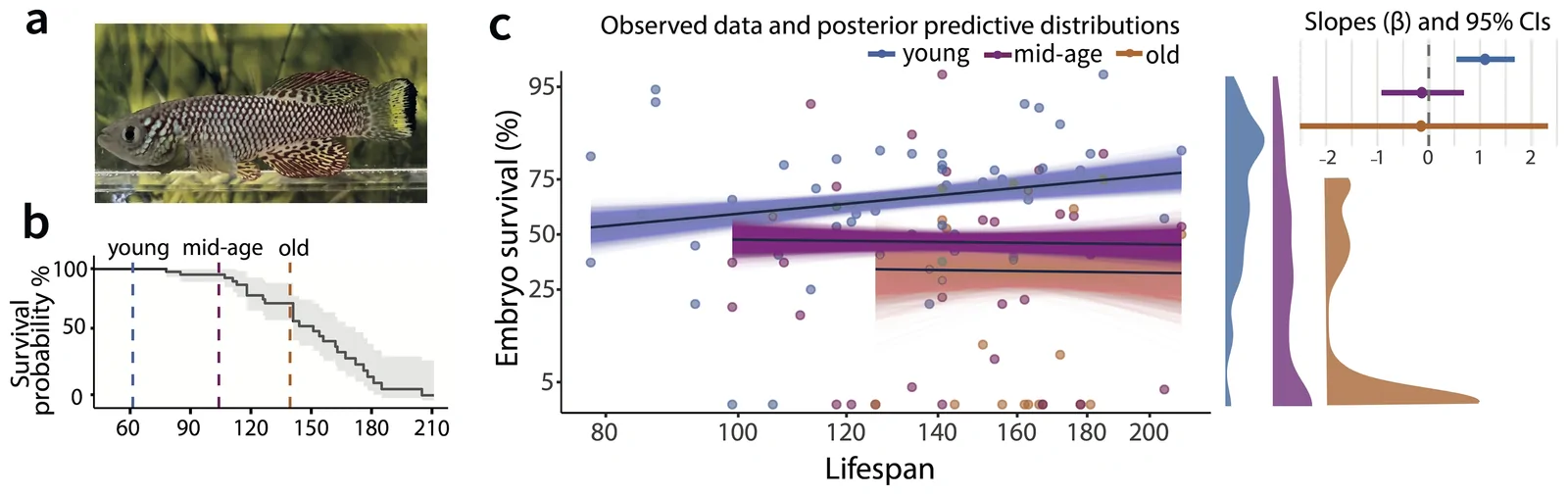
Male reproductive success decreases with age and does not trade off with lifespan. (a) Typical adult male from the GRZ-D strain (photo: S. Cattelan), and (b) survival curves of the male cohort used in the study (median lifespan: 154 days). At three age classes (young, mid-age and old) across males’ lifespan, we collected the ejaculate and performed IVFs. We measured embryo survival, that is proportion of surviving embryos at 72 hours after IVF. Lifespan of each male was also recorded. (c) On the left, a scatterplot showing the relationship between lifespan and embryo survival across the three age classes. Dots represent observed data from each individual male. Mean posterior predictions (black lines) and posterior predictive distributions (coloured lines) from Bayesian models are fitted. Embryo survival was modelled as a binomial outcome with a logit link, and log-transformed lifespan was added as a predictor in the model. For visualization purposes, observed data for embryo survival and lifespan are transformed using the logit function and the log scale, respectively. In the centre, a Kernel density plot depicting embryo survival at young (mean ± s.d.: 63.2% ± 24.3%), mid (mean ± s.d.: 40.4% ± 29.6%) and old (mean ± s.d.: 26.6% ± 31.0%) age. Pairwise comparisons are significant (*p* < 0.001) for all groups except between mid and old age. On the right, a forest plot of slopes (*β* estimates) for each age class. Points indicate posterior means and horizontal bars represent 95% credible intervals, relative to zero as the null effect.

### (b) Early life reproductive success predicts individual lifespan

Because father survival and embryo survival covary for measures taken at young father age, we asked whether embryo survival statistically predicts variation in father lifespan across individuals. Running a Bayesian model for each age class with father lifespan as the outcome variable (figure 2a), we found, in line with the previous model, that the effect of early-life reproductive success on lifespan is positive (figure 2b). Specifically, the posterior predictive distribution of the slope (young age: *β*_mean_ = 0.33; 0.035–0.620, 95% credible intervals; figure 2b) indicates a 95% probability that higher embryo survival is associated with increased male lifespan. To estimate the expected father lifespan for a given embryo survival probability (*p*), we extracted the posterior means of the intercept (*α*) and slope (*β*) from the model and calculated the expected lifespan at *p* as follows: lifespan_(_*_p_*_)_ = exp(*α*_mean_ + *β*_mean_ × *p*). For instance, an increase of 50% in embryo survival predicts a 17% proportional increase in expected lifespan (figure 2c). When running a model using reproductive success measured at older ages, the credible intervals associated to the slope of embryo survival on lifespan include zero both at mid (*β*_mean_ = 0.017; −0.202 to −0.229, 95% credible intervals) and old age (*β*_mean_ = 0.052; −0.164 to −0.269, 95% credible intervals), indicating that measuring embryo survival from mid- and old-age fathers provides no information about their lifespan (figure 2b).

**Figure 2.**
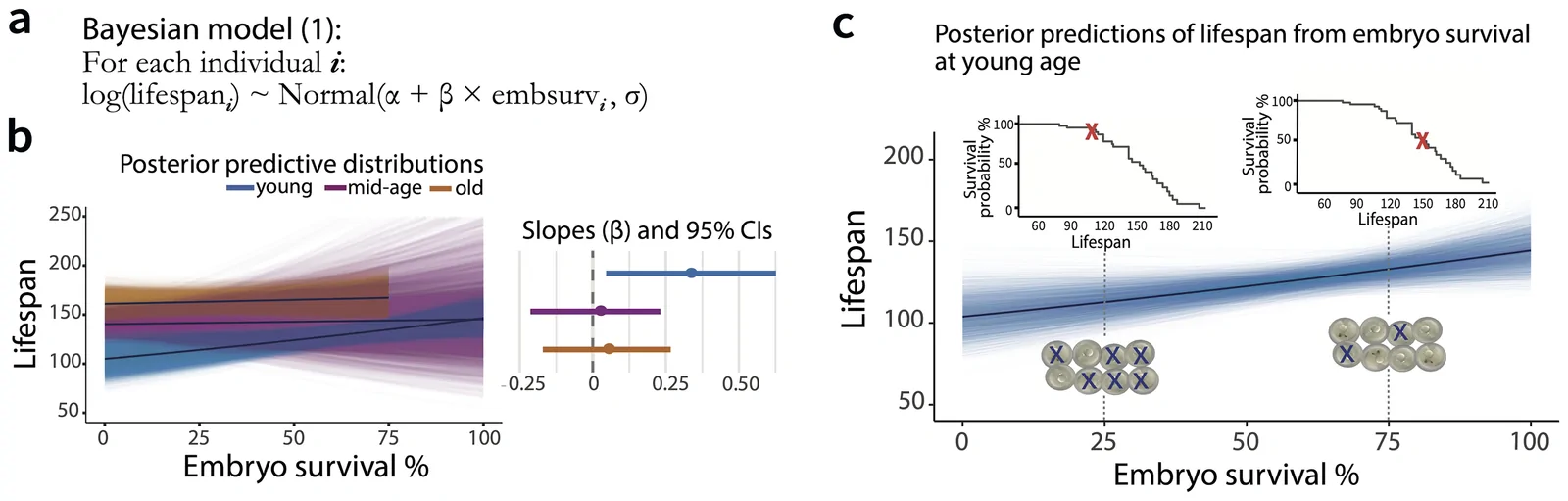
Early life reproductive success predicts individual lifespan. (a) Equation used to run Bayesian models predicting father lifespan from embryo survival. Log-transformed lifespan was modelled as a linear function of embryo survival, where *α* represents the intercept, *β* is the slope (i.e. effect of embryo survival on lifespan) and *σ* is the residual standard deviation. (b) Mean posterior predictions (black lines) and posterior predictive distributions (coloured lines) from Bayesian models run for each age class. Lifespan was modelled as the outcome variable. For visualization purposes, lifespan was back-transformed from the log scale by exponentiation. The equation used to run Bayesian models predicting father lifespan from embryo survival is reported at the bottom. Log-transformed lifespan was modelled as a linear function of embryo survival, where *α* represents the intercept, *β* is the slope (i.e. the effect of embryo survival on lifespan) and *σ* is the residual standard deviation. Each model (one per age class) was run with four chains, each consisting of 2000 iterations, of which 1000 were for warm-up. On the right, a forest plot of slopes (*β* estimates) for each age class. Points indicate posterior means and horizontal bars represent 95% credible intervals, relative to zero as the null effect. At young age, the posterior distribution of the slope indicates a 95% probability that higher embryo survival is associated with increased male lifespan, as the 95% credible intervals do not include 0. (c) Example of posterior predictions for father lifespan at young age. To estimate the expected father lifespan for a given embryo survival probability (*p*), we extracted the posterior means of the intercept (*α*) and slope (*β*) from the model and calculated the expected lifespan at *p* as follows: lifespan_(_*_p_*_)_ = exp(*α*_mean_ + *β*_mean_ × *p*). An increase of 50% in embryo survival (e.g. from 25% to 75%) translates into a 17% increase in lifespan. Lifespan differences are indicated on the survival curves.

### (c) Intrinsic quality explains the age-dependent change in the correlation between individual reproductive success and lifespan

Because trade-off models do not explain the positive covariation between survival and reproduction at the species and individual level, we explored an alternative model, which we formalized as a directed acyclic graph (DAG; figure 3a), a probabilistic approach that helps define causal relationships among variables, identifying potential confounders, mediators and colliders. We modelled that individual quality (hereafter, IQ) causally impacts both individual lifespan and functional quality (FQ). FQ affects the ability to reproduce and thus reproductive success (i.e. embryo survival). Age, in turn, impacts reproductive success both directly and through FQ (figure 3a). This simple model leads to three important consequences. First, variation in IQ among individuals leads to the positive correlation between survival and reproduction. Second, age negatively impacts embryo survival—but not lifespan!—hence, leading to the loss of covariation between reproductive success and lifespan at older age. Furthermore, individuals with low IQ die at young age, reducing the remaining variation of IQ at the population level in middle and old age. Finally, because early-life reproduction is a *bona fide* proxy for IQ, it can be reliably used to predict both parental lifespan and, since it captures both survival and reproduction, Darwinian fitness. Similar to our observed data, the model specified in the DAG showed no negative correlation between embryo survival and lifespan (figure 3b), indicating that the quality-based mechanism is sufficient to reproduce the observed pattern, while not excluding alternative latent-variable formulations of individual heterogeneity. Moreover, simulated individuals with higher embryo survival at young age live longer, whereas embryo survival at mid and old age does not correlate with lifespan (figure 3b), meaning that longer-lived individuals have higher early-life embryo survival compared with individuals living shorter (figure 3c). Because the simulated data produced from our generative model recapitulates our observed data, we asked whether embryo survival statistically predicts variation in father lifespan across simulated individuals in an age-dependent manner. Running a Bayesian model for each age class on the simulated data, we found, in line with the observed data (see figure 2b), that the effect of embryo survival on lifespan is positive at young age (figure 3d). Specifically, the credible intervals associated with the slope of embryo survival on lifespan include zero at young age, but neither at mid nor old age (figure 3d). Consistent with our observed data, our generative model confirmed that the positive correlation between reproduction and lifespan is lost at older ages. Overall, these results indicate that (i) inter-variation in IQ is sufficient to generate the positive correlation between reproduction and lifespan, and (ii) age weakens the correlation between reproduction and lifespan at older ages.

**Figure 3.**
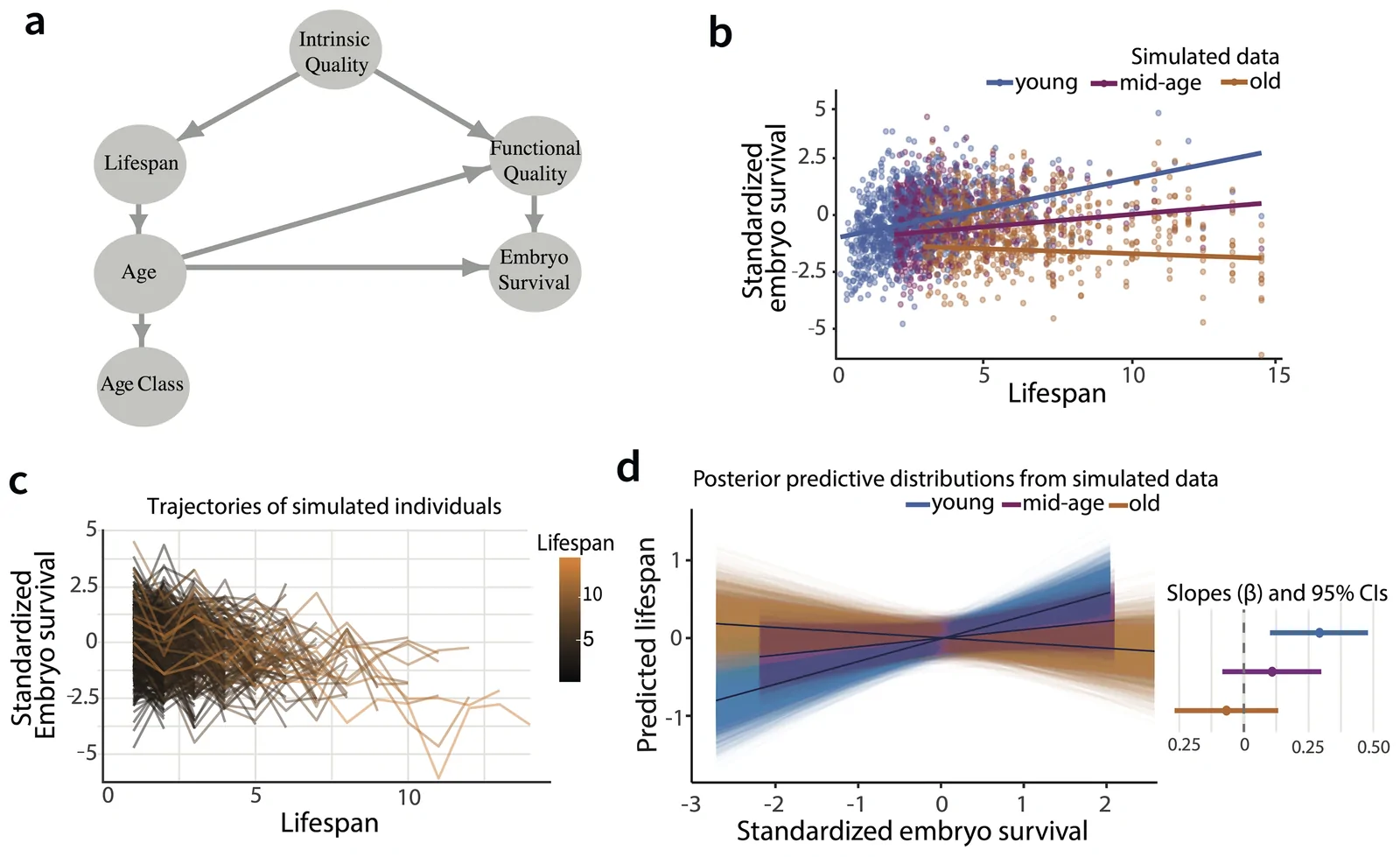
IQ explains the age-dependent change in the correlation between individual reproductive success and lifespan. (a) DAG showing the causal relationships among variables. IQ influences both lifespan and FQ, which in turn affects reproductive success (e.g. embryo survival). Age impacts reproductive success both directly and indirectly through FQ. Longer-lived individuals reach later ages compared with short-lived individuals, making lifespan collinear with age class (e.g. young, mid-age, old). (b) Scatterplot showing the correlation between embryo survival and lifespan in a simulated population in which individuals differ for their IQ. Lifespan and embryo survival were expressed as linear functions of IQ, and age classes were defined according to experimental data thresholds. Each dot represents an individual. Similarly, to the observed data, simulated individuals with higher embryo survival at young age live longer, whereas embryo survival at mid and old age does not correlate with lifespan. (c) Individual trajectories of the relationship between embryo survival and lifespan across ages from simulated data. Each line represents an individual. Individuals with high embryo survival at young age tend to live longer (orange lines) compared with individuals with low embryo survival at young age (black lines). (d) Mean posterior predictions from Bayesian models on the simulated data for each age class. Consistent with observed data, embryo survival at young age but not at mid- and old-age predicts individual lifespan, in accordance with the observed data. On the right, a forest plot of slopes (*β* estimates) for each age class. Points indicate posterior means, and horizontal bars represent 95% credible intervals, relative to zero as the null effect.

## 3. Discussion

Our longitudinal experimental design revealed that males with higher early-life reproductive success also lived longer than those with lower early-life reproductive success. The absence of a clear trade-off between reproduction and survival at the individual level also rules out the possibility that reproductive ageing in killifish arises as a statistical artefact of the selective disappearance of short-lived, high-investing males. These results align with emerging evidence from females of several species, where positive associations between early-life reproduction and survival—rather than trade-offs—define the primary axis of individual variation [29]. For example, in ungulate populations of bighorn sheep, females producing more lambs tend to live longer [11]. Similarly, the number of eggs produced in early life by female killifish positively covaries with lifespan [23].

Growing evidence from humans indicates male infertility as an indicator of poor health and/or lifestyle [30], which is associated with both an increased risk of certain diseases, such as cancers, metabolic and cardiovascular diseases [31] and early mortality [32]. Moreover, a recent meta-analysis found that adult men with high motile sperm count live significantly longer than men with low motile count [33], suggesting that ejaculate parameters can serve as a potential biomarker for long-term survival. Accordingly, we found that higher early-life embryo survival predicts increased male lifespan, indicating that reproductive success early in life can be used to predict lifespan.

In accordance with theoretical and empirical studies, we propose that the positive covariation between reproduction and lifespan reflects intrinsic differences in individual quality (as defined in the introduction). Direct measurement of phenotypic correlates of IQ (sperm motility, morphology, concentration or somatic condition indices) and experimental manipulation of reproductive effort would help distinguish among the specific mechanisms (genetic, developmental, physiological or condition-dependent) by which inter-individual variation in quality could arise. High-quality individuals can simultaneously achieve higher reproductive success and extended survival. The variation in IQ among males is sufficient to explain the loss of correlation between reproduction and lifespan at older ages: as low-quality individuals die earlier, the remaining population becomes increasingly homogeneous in quality, a process known as selective disappearance [34,35]. Moreover, male reproductive senescence, that is, the observed decline in embryo survival with male age, probably contributes to weakening the association between reproduction and lifespan. Simulations based on our DAG model recapitulated the main empirical findings, suggesting that IQ provides a common source of heterogeneity shaping both lifespan and embryo survival.

Beyond our immediate findings, these results inform how lifespan varies within populations. Although the turquoise killifish is a major model for ageing and lifespan evolution among species and population [24,36,37], little is known about drivers of individual lifespan differences. Our data indicate that part of this variation reflects differences in individual quality rather than life-history trade-offs. This suggests that early-life reproductive traits, such as embryo survival or ejaculate quality, could serve as predictors of both healthspan and lifespan. Whether differences in IQ among individuals are genetic, and thus potentially subjected to selection, and how such variation is maintained within populations remain open and exciting questions for future research.

More broadly, our results highlight how life-history trade-offs, while useful and intuitive [38], might not fully explain life-history relationships observed within or between populations. The problem of ‘missing’ trade-off is not new [39], and our results support the current expanded framework of life-history theory that integrates variation in individual quality, ecological and demographic factors, diversity in life stages duration and stochasticity [1,29,40]. By integrating reproductive performance and survival within a unified quality-based model, our results provide a basis for evaluating how reproduction and ageing covary, with implications for understanding the evolution of ageing and life-history diversity.

## 4. Material and methods

### (a) Fish husbandry

The turquoise killifish used in this study were laboratory-reared fish from the GRZ-D strain maintained at the Fish Facility of the Leibniz Institute on Aging (Jena, Germany) under standard laboratory conditions (12 : 12 light : dark cycle; water temperature 26°C ± 1°C; pH 6.5–7.5). Fish were maintained in 3.2 l tanks (density 1.6 l fish^−1^, Aqua Schwarz recirculating system) provided with enrichments. Fish were fed ad libitum twice per day with live brine shrimps (*Artemia salina*) until week 10. From week 4, fish were also fed with bloodworm larvae (*Chironomus* spp.) once per day throughout their life. Extending details on housing and breeding conditions are described in a previous publication [41]. The experiment was carried out under the permission of the following animal experimental license: FLI-22-107.

### (b) *In vitro* fertilizations

To investigate how investment in reproduction affects lifespan, we performed IVFs at three different time points throughout male's lifespan. The average age of males was 62.8 ± 6.2 days (mean ± s.d.) at young age, 103.5 ± 6.8 days at mid-age and 140 ± 4.7 at old age. Between young and mid-age *n* = 8 (approx. 16%) males died, whereas between mid and old age *n* = 20 (approx. 50% of the males alive until that point) males died. The males that survived until old age were *n* = 20 (approx. 41% of the initial males). The median lifespan of the males was 154 days (141–176, 95% confidence interval). Before IVF, we standardized recent social/ sexual history by placing each experimental male with two females (age ranging between 80 and 110 days) in a 10 l tank (Acqua Schwarz), and we let them mate for 5 days. To ensure the replenishment of sperm reserves after mating, each male was isolated from females for 5 days. The male had visual and olfactory access to a female without the possibility to mate with her. On day 10, we performed IVFs by following the protocol that we published previously [42]. To collect sperm from the males, each male was anesthetized in a water bath containing Tricaine (200 mg l^−1^) and then placed on a moist sponge under a stereomicroscope (Leica S9D). After drying the urogenital area with a tissue, sperm were collected using a 5 µl microcapillary by gently pressing the male's abdomen. The ejaculate was then placed in 35 µl Hank's buffer solution and maintained in ice and used within 20 minutes from collection. To collect eggs from the females, each female was anesthetized as above, then dried with a tissue and placed on a standard microscope slide. Eggs were collected by pressing the female's abdomen with an angled chisel sharper. Eggs were then placed in a Petri dish and immersed in a 4% bovine serum albumin (BSA) solution at room temperature until use. For each IVF trial, we pooled eggs from at least 3–4 females to limit the effect of female quality on fertilization rate and embryo survival (mean ± s.d. number of eggs/IVF trial: 33.1 ± 9.16). Immediately before the IVF, we removed the BSA with a disposable Pasteur pipette, and 30 µl of the sperm solution was mixed with 120 µl of tank water and then added to the eggs. The remaining 5 µl of the sperm solution was used to measure sperm concentration using a cell counter (TC20, Bio-Rad). After 5 minutes from the IVF, eggs were immersed in a diluted solution of methylene blue (1 : 1000) and incubated at 28°C. We performed a total of 108 IVF trials (*n* = 48 at young age, *n* = 40 at mid-age, *n* = 20 at old age) using 72 females in total. The age of the females was maintained constant across the experiment (mean ± s.d. female age: 98.2 ± 7.60 days). The same female was used for a maximum of two IVFs with a two week break between the trials.

### (c) Fertilization rate and embryo survival

After 3.5 hours post-IVF, we checked the fertilization rate by counting the fertilized and unfertilized eggs under a stereomicroscope. Fertilized eggs are scored by the presence of dividing cells (two- to four-cell stage). Unfertilized eggs were removed, and the methylene blue solution was changed. The survival of the embryos was then scored 72 hours post-fertilization (hpf) by counting the number of embryos still alive. We scored embryo survival at 72 hpf, which corresponds to the end of the epiboly stage [43], because our previous study revealed that 85% of total embryo mortality occurs within this time frame [22]. In an independent pilot experiment, we found a strong positive correlation (*r* = 0.50, *p* < 0.001) between embryo survival following IVFs and natural matings, suggesting that IVF-derived embryo mortality is not an artefact of the procedure but reflects a biological effect.

### (d) Effect of male age on reproductive success and correlation with lifespan

We ran the following analyses using R v 4.2.0 [44]. To test the effect of male age on embryo survival, we fitted a generalized linear mixed-effect model using the ‘glmer’ function in the ‘lme4’ package [45] and assumed a binomial error distribution and a logit link function. We fitted the model with age class (levels: young, mid-age and old) as a categorical fixed effect, sperm concentration as a continuous fixed effect (i.e. covariate) and male ID as a random effect to account for the non-independence of the data. The model was checked for overdispersion by using the ‘dispersion_glmer’ function within the ‘lme4’ package and corrected by including an observation-level random effect. We calculated *p-*values of the fixed effects by Type II Wald chi-square tests using the ‘Anova’ function in the ‘car’ package [46]. To calculate pairwise effects among the different age classes (young versus mid-age; mid-age versus old; young versus old), we performed post hoc analyses of contrasts with the ‘lsmeans’ function in the ‘emmeans’ package [47] using the Tukey method adjusted for multiple comparisons. The same statistical tests were also applied to fertilization rate (see the supplementary material).

### (e) Bayesian models on observed data

To estimate the parameters that fit the observed relationship between lifespan and reproductive success, we used a Bayesian approach in Stan [28] via the R interface ‘rstanarm’ [27]. For each age class (levels: young, mid-age, old), we ran a Bayesian model to assess whether embryo survival at a specific age was associated with male lifespan. We modelled the number of fertilized eggs that survived 72 hpf as a binomial outcome with a logit link function. The log-odds of embryo survival were modelled as a linear function of the log-transformed lifespan of the father. The model included an intercept (*α*) and a slope (*β*) with weakly informative priors (see equation (4.1)). Each model was run with four chains, each consisting of 2000 iterations, of which 1000 for warm-up:

(4.1) $$\begin{array}{c} \text{for}\;\text{each}\;\text{individual}\;i:\\ n\_\mathrm{embsur}v_{i}∼\mathrm{binomial}\_\mathrm{logit}\left(n\_\mathrm{fertilize}d_{i},\alpha +\beta \times \log(\mathrm{lifespa}n_{i}\right))\\ \mathrm{priors}:\alpha ∼\mathrm{normal}\left(0,\;10\right);\beta ∼\mathrm{normal}\left(0,\;2.5\right), \end{array}$$

where: *n*_embsurv_*i*_ = number of embryos survived for individual *i;n*_fertilized*_i_* = number of fertilized eggs for individual *i*; *α* = intercept; *β* = slope (effect of log-lifespan on embryo survival); and log(lifespan_*i*_) = log-transformed lifespan of individual *i.* We performed similar models for fertilization rate by modelling the number of eggs that were fertilized as the binomial outcome. We assessed model convergence using visual inspection of trace plots (‘traceplot’ function) and checked whether all parameters had R-hat values less than 1.01, indicating good convergence. Posterior probabilities of the intercept and slope were extracted (‘extract’ function) for each age class, and the posterior predictions of embryo survival were computed across a regular sequence of 100 father log-lifespan values. We plotted individual posterior predictions and the mean posterior prediction for each age class using the ‘ggplot2’ package [48] (see associated code).

To estimate the predictive power of embryo survival for father lifespan, we ran a Bayesian model for each age class with lifespan as the outcome variable. In these models, the log-transformed lifespan was modelled as a linear function of the proportion of embryos that survived 72 hpf. The model assumed normally distributed residuals and included an intercept (*α*), a slope (*β*) and a residual s.d. (*σ*), and weakly informative priors (see equation (4.2)). As before, each model was run with four chains, each consisting of 2000 iterations, of which 1000 for warm-up:

(4.2) $$\begin{array}{c} \mathrm{for}\;\mathrm{each}\;\mathrm{individual}\;i:\\ \log\left(\mathrm{lifespa}n_{i}\right)∼\mathrm{normal}\left(\alpha +\beta \times \mathrm{embsur}v_{i},\;\sigma \right)\\ \mathrm{priors}:\alpha ∼\mathrm{normal}\left(0,\;10\right);\;\beta ∼\mathrm{normal}\left(0,\;2.5\right);\;\sigma ∼\mathrm{exponential}\left(1\right), \end{array}$$

where: log(lifespan_*i*_) = log-transformed lifespan of individual *i;* embsurv = proportion of embryos survived for individual *i; α* = intercept; *β* = slope (effect of embryo survival on log-lifespan); *σ* = residual standard deviation. We performed similar models for fertilization rate by using the proportion of fertilized eggs. We assessed model convergence as before (see associated code). As before, posterior probabilities of the intercept and slope were extracted (‘extract’ function) for each age class, and the posterior predictions of lifespan were computed across a regular sequence of 100 embryo survival values. As before, we plotted individual posterior predictions and the mean posterior prediction for each age class using ‘ggplot2’ (see associated code). The same statistical procedures were also applied to fertilization rate (supplementary material).

### (f) Simulations based on the directed acyclic graph

To illustrate whether the hypothesized causal model (DAG, figure 3a) is sufficient to reproduce the observed patterns between lifespan and embryo survival, we used simulated data. The simulation is a single deterministic realization of the generative process specified by the DAG, given the parameters reported below and the random seed used to draw IQ values; it is intended as a forward computation of the DAG's empirical implications, not a Monte Carlo estimate or frequentist hypothesis test on synthetic data. We generated a population of 1000 individuals, each assigned an IQ produced from a normal distribution (mean = 0, s.d. = 1). Lifespan was then simulated for each individual as a log-normal transformation of IQ, whereas FQ was modelled to decline linearly as age increases. At each age (i.e. from 1 until the end of the individual's lifespan), we generated embryo survival from a normal distribution with mean determined by FQ and age. Age classes were defined according to population survival thresholds, consistent with the experimental data: ‘young’ when 85% or greater of the population was alive, ‘mid-age’ between 85% and 60% and ‘old’ when 60% or less of the population was left.

We ran Bayesian models in Stan by, similarly to equation (4.2), modelling lifespan independently for each age class as a linear function of *z*-transformed embryo survival. As before, each model was run with four chains, each consisting of 2000 iterations, of which 1000 were for warm-up. We assessed model convergence as above. Posterior probabilities of the intercept and slope were extracted (‘extract’ function) for each age class, and the posterior predictions of lifespan were computed across a regular sequence of 100 embryo survival values. We plotted individual posterior predictions and the mean posterior prediction for each age class using ‘ggplot2’.

## Supplementary Material



## Data Availability

All data generated are submitted as supplementary material. Supplementary material is available online [49].

## Theme

One contribution of 21 to a theme issue ‘Developmental circuits in ageing’.
