# Supplementary material for: Early-life reproductive success predicts lifespan: intrinsic quality, not reproductive trade-offs, explains heterogeneity in a short-lived vertebrate
Source: Philos Trans R Soc Lond B Biol Sci. 2026 Nov 12;381(1963):20250324. doi: 10.1098/rstb.2025.0324 (PMC13755907; doi:10.1098/rstb.2025.0324)
Supplement: Supplementary file 1 [file rstb.2025.0324.zip › rstb.2025.0324/rstb20250324_si_001.pdf]

Supplementary Results

Early-life reproductive success predicts lifespan: intrinsic quality, not reproductive trade-offs, explains heterogeneity in a short-lived vertebrate

Silvia Cattelan[1] and Dario Riccardo Valenzano[1,2,3]

[1]Leibniz Institute on Aging, Fritz-Lipmann Institute, FLI, Jena, Germany

[2]University Clinic, Friedrich Schiller University, Jena, Germany

[3]Cluster of Excellence Balance of the Microverse, Jena, Germany

Institute on Aging, Jena, Germany; [2]Friedrich Schiller University, Jena, Germany

Supplementary Results

Male fertilization rate decreases with age and does not trade-off with lifespan

To investigate how investment in reproduction affects lifespan, we performed in vitro fertilizations (IVFs) at three different time points throughout male's lifespan. As a measure of reproductive success we recorded fertilization rate and embryo survival. Specifically, after 3.5 hours post IVF we checked the fertilization rate by counting the fertilized and unfertilized eggs under a stereomicroscope. The survival of the embryos was then scored 72 hours post fertilization (hpf) by counting the number of embryos still alive. Here we present the results for fertilization rate (i.e. the number of fertilized eggs on the total number of eggs).

In line with previous work, we found that age significantly affected fertilization rate (χ^2=56.5612, p=5.223e-13). Moreover, sperm concentration significantly affected fertilization rate: males producing more sperm fertilize more eggs (χ^2=7.0762, p=0.0078). Old males fertilized significantly less eggs than young males (z=7.167, p<0.0001) (**Figure S1a**). Similarly to embryo survival, fertilization rate, significantly declines already at mid-age (z=4.919, p<0.0001) (**Figure S1a**).

To test for reproduction-survival trade-offs, we estimated posterior predictive distributions of the relationship between lifespan and fertilization rate at the three different ages (**Materials and Methods**). We found no negative correlation between fertilization rate and individual lifespan (**Figure S1b**). Posterior estimates indicate that males achieving higher fertilization rate at young age live longer

(β_{mean}=0.906; 0.48 – 1.31, 95% credible intervals), while there is no correlation between individual survival and fertilization rate when sampling males at middle (β_{mean}=0.14; -0.38 – 0.31, 95% credible intervals) and old age (β_{mean}=-0.61; -1.98 – 0.84, 95% credible intervals) (**Figure S1c**).

Early fertilization rate predicts individual lifespan

Since father survival and fertilization rate covary, despite weakly, for data collected at young father age (**Figure S1b**), we asked whether fertilization rate statistically predicts variation in father lifespan across individuals. Running a Bayesian model for each age class with father lifespan as the outcome variable (**Materials and Methods, Figure S1d**), we found – as expected from the previous model – that the effect of early-life fertilization rate on lifespan is positive (**Figure S1d**). Specifically, the posterior predictive distribution of the slope at young age (β_{mean}=0.34; 0.006 – 0.69, 95% credible intervals) indicates a 95% probability that higher fertilization rate is associated with increased male lifespan (**Figure S1e**). On the contrary, running a model using fertilization rate measured at older ages, the credible intervals associated to the slope of fertilization rate on lifespan include zero both at mid (β_{mean}=0.037; -0.49 – 0.52, 95% credible intervals) and old age (β_{mean}=-0.13; -0.60 – 0.35, 95% credible intervals), indicating that measuring fertilization rate from mid and old age fathers provides no information about their lifespan (**Figure S1e).**

Figure S1. Fertilization rate largely mirrors the patterns observed in embryo survival. a) Boxplot and Kernel density plot depicting fertilization rate at young, mid and old age. All pairwise comparisons are significant. **b)** Scatterplot showing the relationship between lifespan and fertilization rate across the three age classes. Dots represent observed data from each individual male. Mean posterior predictions (black lines) and posterior predictive distributions (coloured lines) from Bayesian models are fitted. For visualization purposes, observed data for fertilization rate and lifespan is transformed using the logit function and the log scale, respectively. **c)** Forest plot of β estimates for each age class. Points indicate posterior means and horizontal bars represent 95% credible intervals, relative to zero as the null effect. **d)** Mean posterior predictions (black lines) and posterior predictive distributions (coloured lines) from Bayesian models run for each age class. Lifespan was modelled as the outcome variable. For visualization purposes, lifespan was back-transformed from the log scale by exponentiation. Black lines represent posterior means. **e)** Forest plot of β estimates for each age class. Points indicate posterior means and horizontal bars represent 95% credible intervals, relative to zero as the null effect.

Figure S1

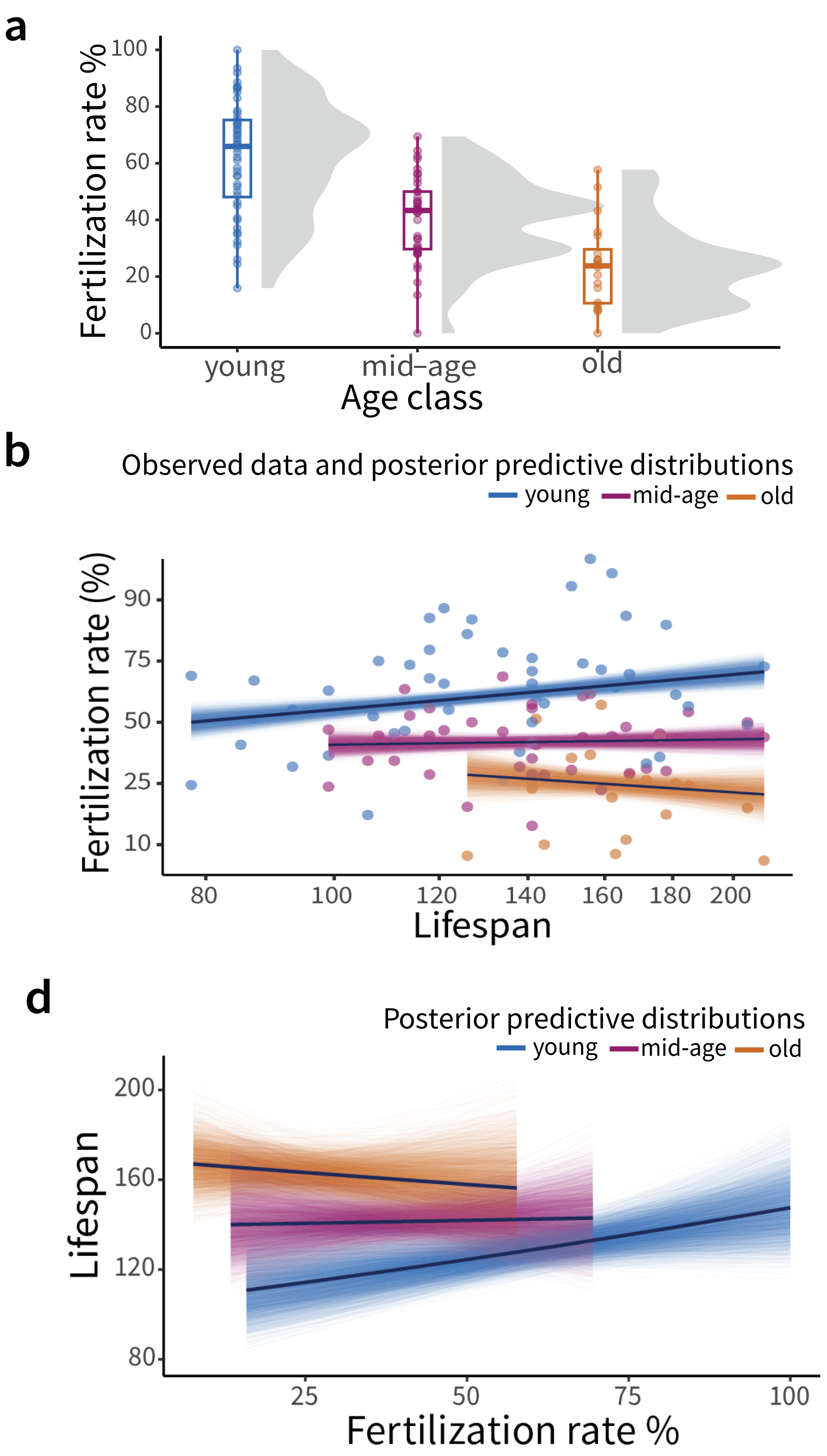

a
Fertilization rate %
100
80
60
40
20
0
young
mid–age
old
Age class
b
Observed data and posterior predictive distributions
young
mid-age
old
Fertilization rate (%)
90
75
50
25
10
80
100
120
140
160
180
200
Lifespan
c
Slopes (β) and 95% CIs
Young
Mid-age
Old
-2
-1
0
1
d
Posterior predictive distributions
young
mid-age
old
Lifespan
200
160
120
80
25
50
75
100
Fertilization rate %
e
Slopes (β) and 95% CIs
Young
Mid-age
Old
–0.4
0
0.4
